# Supplementary material for: FastqPuri: high-performance preprocessing of RNA-seq data
Source: BMC Bioinformatics. 2019 May 3;20:226. doi: 10.1186/s12859-019-2799-0 (PMC6500068; doi:10.1186/s12859-019-2799-0)
Supplement: Supplementary file 2 — Archive of FastqPuri. Archive containing all files needed to install and run FastqPuri v1.0.6. Date stamp March 22, 2019. (GZ 47,819 kb) [file 12859_2019_2799_MOESM2_ESM.gz › FastqPuri-1.0.6/html/struct__tree.html]

FastqPuri: \_tree Struct Reference


FastqPuri

Public Attributes |
List of all members

\_tree Struct Reference

structure containing a T\_ACGT-tree.
More...

`#include <tree.h>`

Collaboration diagram for \_tree:

[legend]

|  |  |
| --- | --- |
| Public Attributes | |
| uint32\_t | L |
|  | |
| uint32\_t | pool\_count |
|  | |
| uint32\_t | pool\_available |
|  | |
| uint32\_t | nnodes |
|  | |
| Node \*\* | pool\_2D |
|  | |

## Detailed Description

structure containing a T\_ACGT-tree.

The tree structure is stored in a pointer to pointer to Node. We grow the structure on the flight as we need more memory. In the outer direction, we start by allocating NPOOL\_2D pointers to Node. In the inner direction, we allocate NPOOL\_1D Nodes and fill them as we read the fasta file. When all of them are allocated, we allocate again NPOOL\_1D. If NPOOL\_2D pointers to Node are allocated, the outer dimension is reallocated with +NPOOL\_2D extra elements. L is the depth of the tree, pool\_count is the number on Node\* elements used so far, pool\_available is the number of Nodes available in every moment, and nnodes is the total number of nodes filled in. We limit the number of allocated nodes to UINT\_MAX (we cannot count more nodes!).

## Member Data Documentation

## ◆ L

uint32\_t \_tree::L

depth of the tree

## ◆ nnodes

uint32\_t \_tree::nnodes

Number of nodes in the tree

## ◆ pool\_2D

Node\*\* \_tree::pool\_2D

2D pool containing the nodes that form the tree

## ◆ pool\_available

uint32\_t \_tree::pool\_available

Number of empty nodes available in the pool

## ◆ pool\_count

uint32\_t \_tree::pool\_count

Number of elements in the second dimension

---

The documentation for this struct was generated from the following file:

- include/tree.h


---

Generated on Mon Mar 19 2018 23:42:01 for FastqPuri by  

 1.8.14
